# Tenofovir rescue therapy in chronic hepatitis B patients who failed previous nucleoside analogue treatment

**DOI:** 10.1007/s12072-015-9681-6

**Published:** 2015-11-26

**Authors:** Dorota Kozielewicz, Waldemar Halota, Magdalena Wietlicka-Piszcz

**Affiliations:** Department of Infectious Diseases and Hepatology, Faculty of Medicine, Nicolaus Copernicus University in Toruń, ul. Św. Floriana 12, 85 030 Bydgoszcz, Poland; Department of Theoretical Foundations of Biomedical Sciences and Medical Computer Science, Faculty of Pharmacy, Nicolaus Copernicus University in Toruń, ul Jagiellońska 13-15, 85 067 Bydgoszcz, Poland

**Keywords:** Antiviral therapy, Chronic hepatitis B, Entecavir resistance, Lamivudine resistance, Tenofovir disoproxil fumarate

## Abstract

**Background:**

Tenofovir (TDF) is considered as the first line therapy for chronic hepatitis B. This study presents the results of TDF monotherapy in patients who failed previous nucleoside analogue treatment.

**Methods:**

The study included 29 patients treated with TDF 245 mg once daily for 18 months after lamivudine monotherapy (LAM arm: *n* = 15) or sequential therapy with lamivudine and entecavir (LAM → ETV arm: *n* = 14). The previous antiviral therapy was discontinued due to lack of efficacy. All patients had HBV DNA between 2.1 and 8.23 log_10_ IU/ml and 15 were HBeAg-positive, while 45 % of patients had increased ALT activity. Undetectable HBV DNA (<20 IU/ml) at months 3, 6, 12 and 18 was the primary endpoint in the study, while HBeAg/HBsAg loss/seroconversion and ALT normalisation were secondary endpoints.

**Results:**

Primary nonresponse to TDF was not observed. HBV DNA was undetectable in 80, 80, 80 and 93 % in LAM arm and 50, 71, 86 and 86 % in LAM → ETV arm patients, at 3, 6, 12 and 18 months of TDF therapy, respectively. One patient achieved anti-HBeAg seroconversion. 86.5 % of patients had normal ALT activity at the end of the study. The baseline HBV DNA load, HBeAg status and the length of the duration of TDF therapy appeared significantly associated with the response to the therapy. HBV DNA clearance occurred faster in HBeAg-negative patients than in those positive for HBeAg.

**Conclusions:**

TDF is an effective antiviral medication in patients with previous exposure to LAM or LAM and ETV. Final proportion of patients who achieved undetectable HBV DNA and had normal ALT activity in both arms, was similar.

## Introduction

Hepatitis B virus (HBV) infection is considered a major health care problem because occult hepatitis can lead to liver fibrosis and subsequent cirrhosis resulting in end stage liver disease. It has been documented that HBV infection is a risk factor for the development of hepatocellular carcinoma [1]. For these reasons, many antiviral therapies have been developed to stop viral replication. Interferon was the first treatment with proven efficacy against HBV. The next lines of therapy were nucleos(t)ide analogues: lamivudine (LAM), adefovir, telbivudine, entecavir (ETV) and recently tenofovir (TDF). A new antiviral medication is being introduced into clinical studies and marketed every few years.

TDF is currently considered as the first-line therapy in chronic hepatitis B patients, regardless of hepatitis B e antigen (HBeAg) status. It is recommended for the treatment of naïve patients but it can also be used in patients who remained HBV DNA-positive after previous antiviral therapies [1]. The number of patients who did not respond to antiviral nucleos(t)ide therapy is growing around the world. It is very important to resolve this problem. The aim of our study is to present the results of TDF monotherapy as a rescue therapy in patients who failed previous nucleoside analogue treatment.

## Materials and methods

### Patients

This is a retrospective review of 29 patients (21 males; 8 females) with confirmed HBV infection, without any other liver disease (HCV infection, Wilson disease, alcoholic liver disease, autoimmune liver disease) or HIV infection. Fifteen patients received LAM monotherapy [100 mg orally, once daily for 7–83 months (mean 38)] (LAM arm) and 14 patients received sequential therapy with LAM [100 mg/day for 6–29 months (mean 16)] followed by ETV [1 mg orally, once daily for 8–70 months (mean 32)] (LAM → ETV arm). During the therapy, patients were monitored by a reverse hybridization method using a Line Probe Assay INNO-LIPA HBV DR V2, DR V2/3 from Immunogenetics^®^ (Ghent, Belgium) for the presence of HBV mutations indicating resistance to LAM (rLAM: rtM204V/I, rtL180M, rtV173L) or ETV (rETV: rtT184G, rtS202I/G) (Table 1). When suboptimal viral response or rLAM occurred, LAM was switched to ETV or discontinued. ETV was stopped when suboptimal viral response was observed or rETV was detected.

**Table 1:** Presence of baseline polymerase sequence mutations conferring nucleoside resistance

Resistance mutation	LAM (*n* = 15)	LAM → ETV (*n* = 14)
M204V	4	1
M204V + L180M	8	6
M204V/I + L180M + V173L	1	3
M204V/I + L180M ± T184G ± S202I/G	0	4
Lack of detected mutations	2	0

*LAM* lamivudine, *ETV* entecavir

Between July 2012 and March 2013, patients who failed previous antiviral therapy by achieving only partial virologic response (PVR), defined as a decrease in HBV DNA of more than 1 log_10_ IU/ml but with HBV DNA measured by real-time PCR assay still detectable after 12 months of therapy, or who developed mutations, were switched directly from their preceding therapies to tenofovir disoproxil fumarate (Viread^®^; Gilead Sciences, USA) therapy (245 mg orally, once daily for 18 months). The polymerase sequence mutations using INNO-LIPA HBV DR V2/3 assay were also checked during TDF treatment at month 3, 6, 12 and 18. However, they could be detected only when HBV DNA concentration was higher than 2.7 log_10_ IU/ml (>500 IU/ml).

All patients had liver biopsy assessed according to Scheuer classification [2]. Liver biopsy was performed within 2 years before the start of tenofovir therapy. Mild fibrosis (S1–S2) was present in 20 subjects, and 8/9 patients with advanced fibrosis (S3–S4) had compensated liver cirrhosis.

## Methods

All treatments (LAM, ETV, TDF) administered to patients described in this report and monitoring of treatment efficacy were standard of care procedures performed according to product characteristics, scientific guidelines [1, 3] and local legal regulations.

Patients had a physical examination and laboratory testing at baseline and after 3, 6, 12 and 18 months of TDF treatment. The primary endpoint of the study was undetectable HBV DNA (<20 IU/ml) at month 3, 6, 12 and 18 of treatment. HBeAg/HBsAg (hepatitis B surface antigen) loss/seroconversion and alanine aminotransferase (ALT) normalisation were secondary endpoints.

HBV DNA was quantified by PCR-based method, with product analysis using real-time PCR, automatic DNA isolation on the Cobas AmpliPrep apparatus and amplification on a Cobas TaqMan Roche analyser (Amplicor HBV monitor; Roche Diagnostics, Basel, Switzerland). The lower limit of quantification was 20 IU/ml and it was linear over the range from 20 to 1.7 × 10^8^ IU/ml (1.3–8.23 log_10_).

HBsAg, HBeAg and HBeAb (hepatitis B e antibody) were determined by chemiluminescent immunoassays (CLIA), using a test from Biomedica (Vienna, Austria) and the LIAISON XL analyser from DiaSorin (Saluggia, Italy).

ALT activity was measured using the ICFF method, without pyridoxal phosphate at the temperature of 37 °C, using a test kit from Roche and Cobas Integra analyser (Roche Diagnostics). ALT level ≤33 U/l and ≤41 U/l was considered as normal for female and male, respectively.

Patients were not exposed to any additional activities or study-related risk or procedures. The epidemiological case report review was used to collect the data described in this report and patients’ identities were not disclosed at any point in this report; thus, there was no need to obtain Ethics Committee approval for this study.

### Statistical analysis

The results of TDF monotherapy assessed as undetectable versus detectable HBV DNA level and normal versus increased ALT activity in subsequent months of therapy have been analysed using the generalized estimating equations (GEE) for binary data, which takes an account of the correlation between repeated observations from the same individual at multiple time points. The statistics for continuous variables are presented as median with range and the categorical variables are presented as frequencies. Differences between continuous variables were analysed by Wilcoxon test and differences for categorical variables were tested using the *χ*^2^ or Fisher exact test for independence.

The results were considered statistically significant, when the *p* value was <0.05. The statistical analysis was performed with the use of the R software, v.3.0.3.

## Results

### Baseline patient characteristics

At the beginning of TDF therapy, the patients’ median age was 41 years (20–81) and the median duration of HBV infection was 14 years (1–33). The median viral load was 3.68 log_10_ IU/ml (2.1–8.23 log_10_). Fifteen patients were positive for HBeAg. HBeAg was detectable in 4 patients from the LAM arm and in 11 from the LAM → ETV arm. Median ALT activity in the study group was 28 U/l (12–500). Thirteen patients (45 %) had increased ALT activity (6 in the LAM arm, 7 in the LAM → ETV arm). Except for HBeAg distribution, there were no statistically significant differences between patients from the LAM and LAM → ETV groups at the beginning of TDF treatment (Table 2).

**Table 2:** Baseline characteristics of patients

	Total (*n* = 29)	LAM arm (*n* = 15)	LAM → ETV arm (*n* = 14)
Sex (male/female)	21/8	11/4	10/4
Age (years)			
Min–max	20–81	21–78	21–81
Median	41	43	31
Duration of HBV infection (years)			
Min–max	1–33	1–33	4–22
Median	14	16	12
HBV DNA (log_10_ IU/ml)			
Min–max	2.1–8.23	2.1–8.23	2.21–7.48
Median	3.68	3.10	4.30
HBeAg status*			
Positive	15	4	11
Negative	14	11	3
ALT (U/l)			
Min–max	12–500	12–253	14–500
Median	28	25	33
Liver fibrosis (staging)			
S1–S2	20	11	9
S3–S4	9	4	5
Activity of liver inflammation (grading)			
G0–G2	20	11	9
G3–G4	9	4	5

*LAM* lamivudine, *ETV* entecavir, *ALT* alanine aminotransferase, *HBeAg* hepatitis B e antigen, *HBV DNA* viral load

* χ ^2^: *p* = 0.01

### Virologic response

**Fig. 1:**
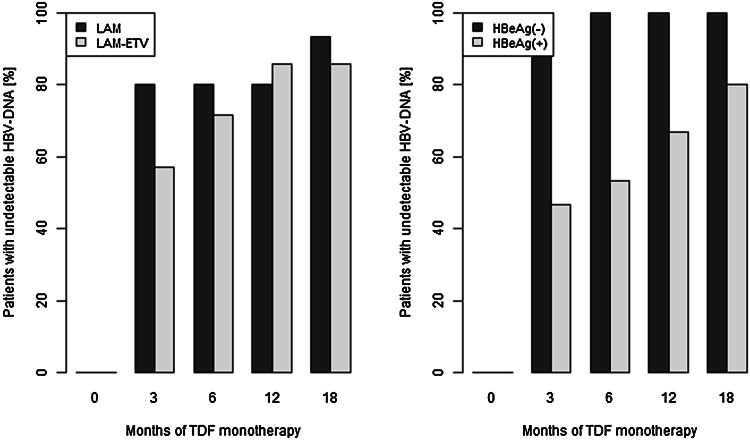
Virologic response during TDF treatment in LAM arm versus LAM → ETV arm (*left*, *p* > 0.05) and in HBeAg-positive versus HBeAg-negative patients (*right*, *p* = 0.0006)

At month 18 of the study, 26 (90 %) patients achieved complete virologic response (CVR), defined as undetectable HBV DNA (<20 IU/ml), measured by real-time PCR assay after 12 months of therapy [1]. The proportion of patients with undetectable HBV DNA in the LAM arm (93 %) did not differ significantly from the fraction of patients in the LAM → ETV arm (86 %) (Fig. 1, left diagram).

In the LAM arm, the proportion of patients with undetectable HBV DNA was 80 % at months 3, 6 and 12, and increased to 93 % at month 18 (Fig. 1, left). In the LAM arm, one patient had only partial virologic response at month 18; however, the viral load was near the lower limit of detection and registered as 1.47 log_10_ (30 IU/ml).

In the LAM → ETV arm, the proportion of patients with undetectable HBV DNA at month 3 was 50 %, but increased to 71 % at month 6 and 86 % at months 12 and 18 (Fig. 1, left). Partial virologic response was achieved in 14 % of patients at month 18 of treatment. The mean HBV DNA level was 1.75 log_10_ (57 IU/ml).

All patients had at least 1 log_10_ reduction in HBV DNA concentration between baseline and 3 months of TDF monotherapy. Primary nonresponse to TDF was not observed. None of the patients developed virologic breakthrough, defined as confirmed increase of HBV DNA ≥ 1 log_10_ IU/ml from nadir [1].

In order to study the dynamics of virologic response to TDF therapy and the association of the response and factors which can increase or decrease the risk of insufficient response to the therapy, a GEE model with logit link function has been applied. The potential influence of HBeAg status, baseline HBV DNA concentration, liver fibrosis, age and gender of the patients, length of the duration of TDF monotherapy as well as the type of previous therapy (LAM vs. LAM → ETV) and the presence of polymerase sequence mutations on response has been checked. However, for the examined group of patients, HBeAg status, baseline HBV DNA concentration and the length of the duration of TDF therapy appeared significantly associated with the response to the therapy (Table 3, Models 1 and 2), while other factors (liver fibrosis, age and gender of the patients, the type of previous therapy and the presence of polymerase sequence mutations) seem not to exhibit a significant influence on the dynamic of virologic response. A higher HBV DNA concentration at baseline was observed, the lower being the probability of virologic response to the therapy. The increase of HBV DNA concentration by 1 log_10_ at baseline decreases the odds of virologic response versus no response by about 70 % on average [OR = 0.29 (0.15 0.56); Table 3, Model 1] at any time point. The length of duration of the TDF therapy increases the probability of achieving undetectable HBV DNA and the passage of each month increases the odds of undetectable HBV DNA by about 20 % on average [OR = 1.22 (1.00 1.48); Table 3, Model 1]. HBeAg status at baseline also affected the virologic response and HBeAg-positive status decreases the odds of the loss of HBV DNA [OR = 0.25 (0.11 0.55); Table 3, Model 2; Fig. 1, right].

**Table 3:** Results of the estimation of GEE models for virologic response i.e. undetectable versus detectable HBV DNA (*Model 1* and *Model 2*) and ALT activity i.e. normal versus increased ALT activity (*Model 3*)

	Estimate	Robust SE	*Z*	*p* value	OR (CI)
GEE models for virologic response (undetectable vs. detectable HBV DNA concentration)					
*Model 1*:					
(Intercept)	5.890	1.578	3.733	0.0002	
Time	0.196	0.100	1.961	0.0499	1.22 (1.00 1.48)
HBV DNA at baseline (log_10_)	−1.250	0.344	−3.631	0.0003	0.29 (0.15 0.56)
*Model 2*:					
(Intercept)	−0.225	0.196	−1.151	0.2496	
Time	0.264	0.076	3.476	0.0005	1.30 (1.12 1.51)
HBeAg-positive at baseline (Ref: HBeAg-negative)	−1.396	0.405	−3.446	0.0006	0.25 (0.11 0.55)
GEE model for ALT activity (normal vs. increased ALT activity)					
*Model 3*:					
(Intercept)	2.341	0.587	3.991	<0.0001	
Time	−0.019	0.037	−0.507	0.6119	0.98 (0.91 1.05)
HBV DNA (log_10_)	−0.528	0.111	−4.748	<0.0001	0.59 (0.47 0.73)

*OR (CI)* odds ratio with 95 % confidence interval, *Robust SE* robust standard error

### Serologic response

HBsAg loss was not observed. All patients remained positive for HBsAg at the end of the study. HBeAg/anti-HBe seroconversion occurred in one patient in the LAM arm at month 18 of treatment.

### Biochemical response

**Fig. 2:**
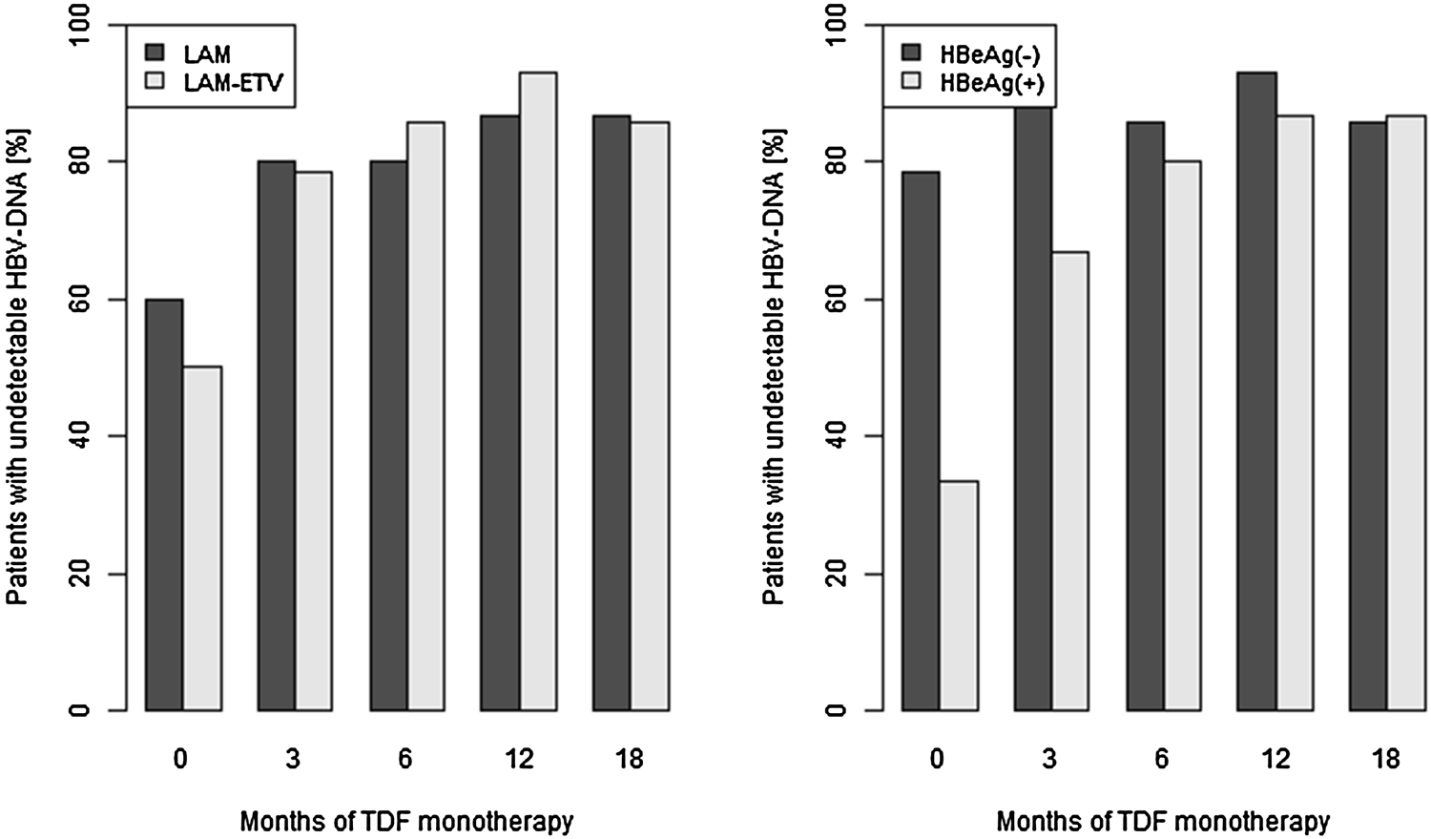
Patients with normal ALT activity in LAM arm versus LAM → ETV arm (*left*, *p* > 0.05) and in HBeAg-positive versus HBeAg-negative patients (*right*, *p* = 0.0381 at baseline)

There were no statistically significant differences between the proportion of patients with normal ALT activity at month 18 between the LAM arm (87 %) and LAM → ETV arm (86 %) (Fig. 2, left). At the end of the observation period, ALT normalisation was observed in 86.5 % of patients.

In Fig. 2 (right), the proportion of patients with normal ALT activity in HBeAg-positive and HBeAg-negative groups is also shown. There was significant difference between the proportion of patients with normal ALT activity in HBeAg-negative and HBeAg-positive group at baseline (11/14 vs. 5/15, *p* = 0.0381), but at month 3 and subsequent months of the therapy, the difference was no longer significant.

In order to investigate the dynamics of ALT activity in subsequent months of therapy, a GEE model has been fitted to the data (Table 3, Model 3). The potential influence of HBeAg status, HBV DNA concentration, liver fibrosis, age and gender of patients, the type of previous therapy (LAM vs. LAM → ETV) and the presence of the polymerase sequence mutations on the increased ALT activity has been evaluated. For this group of patients, HBV DNA concentration (expressed as log_10_ HBV DNA) appeared to be the only factor significantly associated with ALT activity status. A higher HBV DNA concentration was observed, the lower being the probability of normal ALT activity. The increase of HBV DNA concentration by 1 log_10_ decreases the odds of normal ALT activity by about 40 % [OR = 0.59 (0.47 0.73)]. The other considered factors seem not to show significant association with increased ALT activity.

### Polymerase sequence mutations during TDF monotherapy

The presence of polymerase sequence mutations at baseline did not influence virologic response during TDF monotherapy. They were detectable until month 6 of the treatment only in four patients (three in the LAM arm and one in the LAM → ETV arm) and remained the same at all check points. Various combinations were observed; in the LAM arm: M204V, M204V + L180M and M204V + L180M + V173L, each in one patient; in the LAM → ETV arm: M204V + L180M + T184G + S202G.

## Discussion

The approach to the therapy of chronic hepatitis B has been changing according to the availability of new antiviral medications. Interferon was the first antiviral therapy used in Poland, financed from public funds through the therapeutic anti-HBV programme [4]. Lamivudine was introduced later as the first orally taken anti-HBV medication, and subsequently other medications became available [3, 5]. Patients participating in this study had a long history of HBV infection. All subjects in the study had received lamivudine, as for many years it was available as the main antiviral medication in therapeutic programs reimbursed by the Polish health care system.

Lamivudine seemed to have similar efficacy to interferon but had none of the side effects reported with interferon therapy. However, the efficacy of LAM was limited by the emergence of HBV mutations that were resistant to this therapy. LAM resistance increases progressively during treatment at rates of 14–32 % annually, exceeding 70 % after 2 years of treatment [6]. For this reason, patients receiving LAM are tested for HBV DNA load and for the presence of these mutations. Lamivudine should be discontinued or the patient switched to other antiviral treatment if HBV DNA remains unchanged or a mutant variant appears. In our study, the proportion of patients positive for rLAM was greater than 75 % over the mean treatment period of 38 months; thus, the recommended approach to discontinue or switch the antiviral therapy was justified.

Entecavir was introduced into Polish therapeutic programs for HBV patients a few years after lamivudine. It could be used only in sequential therapy in patients who failed LAM. ETV was well tolerated but the efficacy profile was only slightly better than lamivudine and resistance mutations could still occur. Due to its high frequency of virologic breakthrough (43 % after 5 years of treatment), the role of ETV salvage therapy in LAM-refractory patients was limited [7]. The patients analysed in this study were treated according to the Polish anti-HBV therapeutic program regulations. All of them received LAM, then stopped it and were switched to ETV when virologic breakthrough or at least one resistance mutation was detected. Sequential LAM and ETV therapy resulted in a large number of patients with resistance mutations to these medications.

It is well known that long-lasting HBV infection is a slowly progressing process leading to severe liver disease. For this reason, patients with HBV DNA replication are considered candidates for antiviral therapy. The majority of patients analysed in this study had mild liver disease with normal or slightly abnormal ALT activity and mild liver fibrosis. All of them remained highly positive for HBV DNA and thus were at risk of liver disease progression; for this reason, they were considered candidates for further antiviral therapy.

TDF is a nucleotide analogue without cross-resistance with nucleoside analogues like lamivudine, telbivudine and entecavir. It is highly effective in the treatment of naïve patients. Multicentre randomised phase III studies have shown that 7 years of the therapy resulted in undetectable HBV DNA (<400 copies/ml) in 77 % of HBeAg-negative and 60 % of HBeAg-positive patients. ALT normalisation was achieved in 84 and 74 % of these patients, respectively. Forty percent of subjects lost HBeAg [8]. Baran et al. [9] have reported the efficacy of TDF in 92 rLAM patients with high HBV DNA load (7.11 log_10_ IU/ml). Twenty-one patients received TDF monotherapy, while the remaining subjects were treated with a combination of LAM and TDF. At month 24 of therapy, a complete virologic response (HBV DNA < 20 IU/ml) was noted in 89 and 88 % of these patients, respectively. Similar results of TDF efficacy have been reported in the ongoing long-term study (EudraCT no: GS-US-174-0121) comparing TDF monotherapy with TDF and emtricitabine combination in patients with rLAM. At week 96 of therapy, HBV DNA was undetectable (<400 copies/ml) in 89 % of rLAM patients who had baseline HBV DNA load greater than 10^3^ copies/ml [10]. Sangheun et al. [11] reported 70 % rate of complete virological response (HBV DNA < 20 IU/ml) at month 12 of TDF monotherapy in rLAM patients with a mean baseline HBV DNA load of 3.6 log_10_ IU/ml.

Our study confirms the above clinical observations on the efficacy of TDF monotherapy in reducing HBV replication in patients who failed LAM treatment. Complete virologic response at month 18 of therapy was achieved in 93 % of patients who failed previous antiviral therapy with LAM and had a high viral load at the beginning of TDF therapy. The majority of these patients were positive for LAM resistance mutations.

Sequential LAM → ETV treatment has been used in some regions in the past; however, it has never been widely recommended. Thus, the number of patients who have received this type of therapy is small and the number of reports on the efficacy of TDF therapy following the sequential LAM → ETV regimen is limited. Van Bömmel et al. [12] reported one case and Lee et al. [13] three cases of patients treated with TDF after sequential LAM → ETV therapy. There are also reports describing the efficacy of TDF in patients with a suboptimal virologic response during 24 weeks of ETV therapy. Pan et al. [14] described the elimination of HBV DNA and ALT normalization in 100 % of patients resistant to ETV after 30 weeks of TDF treatment. These authors recommend an early switch to TDF, before virologic breakthrough or the emergence of resistance mutations occurs.

Nowadays, TDF is recommended in patients with multi-drug resistance to LAM and ETV [15, 16], and it is estimated that the proportion of patients with HBV DNA clearance (HBV DNA < 20 IU/ml) should reach around 65 % after 12 months of therapy [17]. The results described in this paper show that 86 % of patients reached the goal of the therapy at month 12. A CVR rate of 86 % at month 18 of TDF therapy is satisfactory and is not statistically different from the response in patients treated with LAM monotherapy.

Our results show high efficacy of TDF, regardless of previous antiviral therapy with LAM or LAM → ETV. However, there are several limitations to the study: it is retrospective, the number of patients is small, and the duration of observation is short. Despite these limitations, we confirmed the high efficacy of TDF monotherapy especially in patients with previous ineffective sequential LAM → ETV therapy. Likewise, history of prior exposure (LAM monotherapy vs. sequential LAM → ETV therapy) had no influence on treatment outcome with tenofovir.

On the other hand, the data collected in this study come from everyday clinical practice. The patients had documented HBV replication for many years and TDF was the first antiviral medication that changed their virologic status. Thus, we think that TDF monotherapy may be effectively used in patients who did not respond to previous LAM or LAM → ETV therapy. We demonstrated high efficacy of TDF in reducing HBV DNA load, but only one (7 %) patient was cleared of HBeAg. Our result is close to the 11 % rate of HBeAg clearance observed in the ongoing GS-US-174-0121 study [10], but much lower than that reported by Baran et al. (32 %) [9]. The latter authors reported that HBeAg clearance occurred after at least 15 months of TDF therapy. The patients described in our paper were not treated for longer than 18 months, and the age at which they became infected with HBV did not predispose them to a higher rate of HBeAg seroconversion.

Finally, effective suppression of HBV DNA is usually followed by a reduction of inflammatory activity and normalisation of ALT. This has been observed in all reports [8–11, 17]. Our data show a marked increase in the percentage of patients with normal ALT activity in both groups of patients treated with TDF.

## Conclusion

TDF is an effective anti-HBV medication in patients with previous exposure to LAM or LAM and ETV. Our study showed that HBeAg status, baseline HBV DNA concentration and duration of TDF therapy are significantly associated with the response to therapy. TDF-induced HBV DNA clearance occurred faster in HBeAg-negative patients than in those positive for HBeAg. Furthermore, some HBeAg-positive patients did not achieve undetectable HBV DNA. Finally, the proportion of patients who achieved the primary and secondary endpoints at 18 month of therapy did not differ significantly in both arms.
